# Gross motor developmental dysfunctional outcomes in infantile and toddler pediatric intensive care unit survivors

**DOI:** 10.1186/s12887-019-1893-9

**Published:** 2019-12-21

**Authors:** Chun-Feng Yang, Yang Xue, Jun-Yan Feng, Fei-Yong Jia, Yu Zhang, Yu-Mei Li

**Affiliations:** 1 Department of Pediatrics Intensive Care Unit, The First Hospital of Jilin University, Changchun, 130021 China; 2 Department of Developmental and Behavioral Pediatrics, The First Hospital of Jilin University, Changchun, China

**Keywords:** Gross motor developmental function, Sepsis, Mechanical ventilation, Pediatric intensive care unit, Infant, Toddler

## Abstract

**Background:**

Increasing studies have focused on motor function/dysfunction in PICU survivors; however, most studies have focused on adults and older children. This study investigated gross motor developmental function outcomes in infantile and toddler pediatric intensive care unit (PICU) survivors and the factors associated with gross motor developmental functions.

**Methods:**

This observational study was conducted in the PICU of the First Hospital of Jilin University between January 2019 and March 2019. Thirty-five eligible patients were divided into the dysfunctional (*n* = 24) or non-dysfunctional (*n* = 11) group according to the results of the Peabody Developmental Motor Scales, Second Edition (PDMS-2). Baseline gross motor function for all participants before PICU admission was measured via the Age and Stages Questionnaires, Third Edition (ASQ-3). The PDMS-2 was used to evaluate gross motor development function before PICU discharge.

**Results:**

The gross motor developmental dysfunction incidence was 68.6%. Linear correlation analysis showed that the gross motor quotient (GMQ) was positively correlated with the pediatric critical illness score (PCIS, *r* = 0.621, *P* < 0.001), and negatively correlated with length of PICU stay (*r* = − 0.556, *P* = 0.001), days sedated (*r* = − 0.602, *P* < 0.001), days on invasive mechanical ventilation (IMV; *r* = − 0.686, *P* < 0.001), and days on continuous renal replacement therapy (CRRT; *r* = − 0.538, *P* = 0.001). Linear regression analysis showed that IMV days (β = − 0.736, *P* = 0.001), sepsis (β = − 18.111, *P* = 0.003) and PCIS (β = 0.550, *P* = 0.021) were independent risk factors for gross motor developmental dysfunction.

**Conclusions:**

Gross motor developmental dysfunction in infantile and toddler PICU survivors is more common and may be exacerbated by experiences associated with longer IMV days and increasing illness severity combined with sepsis.

**Trial registration:**

The trial ‘Early rehabilitation intervention for critically ill children’ has been registered at http://www.chictr.org.cn/showproj.aspx?proj=23132. Registration number: ChiCTR1800020196.

## Introduction

With improved diagnostic and treatment technology in pediatric critical care medicine, mortality rates have decreased significantly among critically ill children. However, treatments such as invasive mechanical ventilation (IMV), drugs and other factors, can cause newly acquired functional disabilities, in addition to saving the lives of critically ill children [1–3]. Increasing studies have focused on motor function/dysfunction in PICU survival [1, 4, 5]; however, most have focused on adults and older children. Because motor function in older children is similar to that in adults, the methods mostly assess muscle strength, mobility, fatigue and the activities of daily living scale (ADLS), and most assessment results are obtained via questionnaires [4, 6–8]. However, these assessment methods do not apply to infants and toddlers in the PICU. Because children are not “little adults” [9], their gross motor functions are in the developmental stage, which is a critical period for gaining motor skills. Therefore, assessing gross motor developmental function can better reflect the physical functions of infants and toddlers.

Few studies have focused on acquired gross motor developmental dysfunction in children. Hövels-Gürich et al. [10] found that the neonatal arterial switch operation with combined circulatory arrest and low-flow bypass was associated with neurological and fine and gross motor impairment. In addition, gross motor developmental function assessment is mostly used in neonatal intensive care units (NICUs) and in high-risk infants [11, 12]. Patients are at different risks for having developmental dysfunction between NICU and PICU, therefore, the two populations differ entirely [13, 14]. Therefore, our study investigated the gross motor developmental function outcomes of infantile and toddler survivors of pediatric intensive care units (PICUs) and is the first to assess gross motor developmental function in infants and toddlers in a PICU.

## Materials and methods

### Patients

This observational study was conducted in the pediatric intensive care unit of the First Hospital of Jilin University, ChangChun, China. Eligible cases were children aged between 1 month and 3 years who were hospitalized in the PICU ≥48 h between January 2019 and March 2019, and for whom it was their first PICU admission during the study period. Children were excluded if they had neuromuscular junction disease, central nervous system disease, limb fractures or deep vein thrombosis. Children with gross motor developmental dysfunction before PICU admission were also excluded. The hospital’s ethics committee granted permission for the study, and the eligible children’s parents/guardians provided written informed consent. The trial was registered at clinical trials.gov (ChiCTR1800020196). All participants’ information sheets were provided to their parents.

### Procedure

The PICU cohort was categorized into two groups: the dysfunctional group (GMQ < 90) and the non-dysfunctional group (GMQ ≥ 90). Age, sex, diagnosis, severity of illness, length of PICU stay, days on IMV, days on methylprednisolone, days sedated, days on continuous renal replacement therapy (CRRT), and application of vasoactive drugs were recorded for each group. Because of the sample size, primary diagnoses were broadly categorized as cardiovascular, respiratory, gastrointestinal, or other (genitourinary, hematologic/oncologic, musculoskeletal, endocrinologic, or trauma). Baseline gross motor function was measured for all participants before PICU admission using the Age and Stages Questionnaires, Edition 3 (ASQ-3) to assess the gross motor developmental function of participants prior to PICU admittance. At the time of PICU discharge, all patients completed the Peabody Developmental Motor Scales, Second Edition (PDMS-2), which assesses gross motor development function [15, 16]. To ensure the accuracy of the assessment results, one experienced physiotherapist assessed all participants in an assessment room that met the conditions for motor assessment.

### Measures

Illness severity was measured using the pediatric critical illness score (PCIS) [17] developed by the Chinese Medical Association Emergency Department and the Chinese Medical Association Emergency Society Pediatrics Group. The PCIS is currently the most widely used pediatric critical illness scoring method in China, and it’s total score is negatively correlated with the severity of the disease which can accurately determine the condition and predict the risk of death in children [18].

The ASQ is a reliable, standardized, parent-completed, developmental screening test composed of 21 age-specific questions covering the ages of 1–66 months [19]. And it is a reliable and valid measure which can be used to screen and monitor the development of children in the mainland of China [20]. The ASQ-3 encompasses five developmental areas: communication, gross motor, fine motor, problem solving and personal-social [21, 22]. Three responses are possible per item, depending on whether the child can perform the task: “Yes” (10points), “Sometimes” (5 points) and “Not Yet” (0 points). The total score for each area is obtained by adding the scores of the six items. The assessment results are divided into normal, critical and abnormal based on each area’s total score. Only those children with normal results of ASQ were enrolled in our study.

At the time of PICU discharge, the patients’ gross motor developmental function was measured using the PDMS-2, which is a norm-referenced tool designed to assess the fine and gross motor skills of children aged between 0 and 71 months. Its normative sample was based on 2003 children in 46 states of the United States and one Canadian province [23]. The PDMS-2 is suitable for assessing various populations of children at high risk for motor delays. It has a high degree of reliability and validity regarding child development in China [24–26], but few studies have applied the PDMS-2 to children in PICUs. The PDMS-2 is composed of four subtests: reflex, stationary, locomotion, and object manipulation, and each subtest contributes to the gross motor quotient (GMQ) score. The scores are interpreted as very superior (131–165), superior (121–130), above average (110–120), average (90–109), below average (80–89), poor (70–79), or very poor (35–69) [27]. Gross motor developmental dysfunction was defined as GMQ < 90 [27].

### Statistical analyses

Data were analyzed using IBM SPSS Statistics for Windows, version 22.0 (IBM Corp, Armonk, NY, USA). Continuous variables are described as the mean ± SD or median (interquartile range), depending on whether the distribution was normal or non-normal. Normality test of variables using Shapiro-Wilk test. Categorical variables are described as n (%). Continuous variables were compared using Student’s t-test or the Mann-Whitney U test. Categorical variables were compared using the chi-square test or Fisher’s exact test depending on sample size. For the correlation analyses, the Spearman method was used to test the relationship between GMQ and PCIS, length of PICU stay, IMV days, days on methylprednisolone, days sedated and CRRT days. The relationship among multiple factors was analyzed via multivariate linear regression (stepwise method), and the dependent variable, Y, was a continuous variable with a normal distribution. For all final comparisons, *P* ≤ 0.05 was considered statistically significant.

## Results

### Sample characteristics

**Fig. 1:**
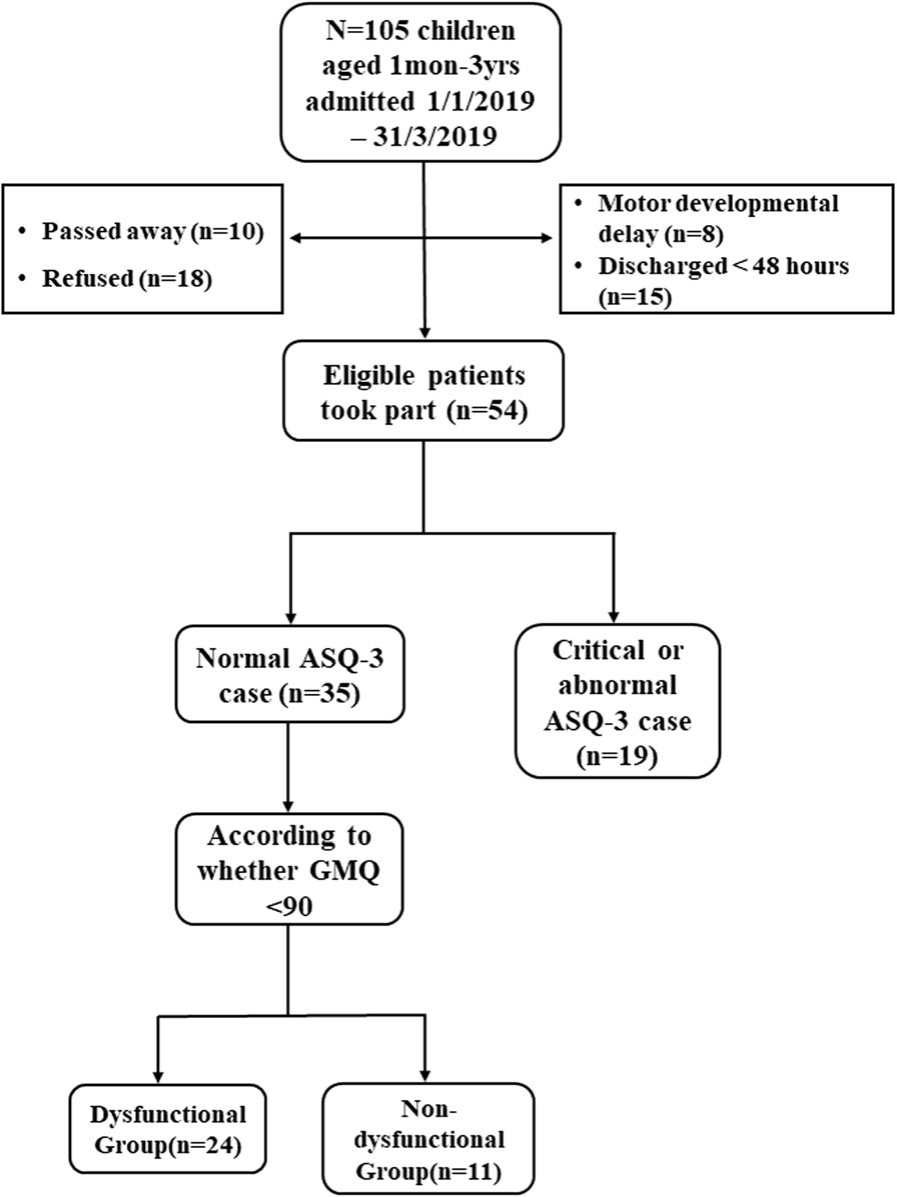
Flow chart for the study

During the study period, 70 of 105 PICU patients were excluded. Of these 70 patients, 8 had motor developmental delays before entering the PICU, 18 had parents who refused to give permission for the motor development assessment, 10 died during PICU hospitalization, 19 had abnormal ASQ-3 results, and 15 were discharged within 48 h of PICU admittance. Finally, 35 patients met the inclusion criteria. Participants were categorized as either children with developmental dysfunction (*n* = 24) or children without developmental dysfunction (*n* = 11) based on whether the GMQ was ≥ or < 90 according to the results of PDMS-2 (Fig. 1).

Age, sex, diagnostic category, percentage of surgical patients, percentage of sepsis, use of vasoactive medications, PCIS, length of PICU stay, IMV days, days on sedatives, days on methylprednisolone and CRRT days were recorded for both groups (Table 1). Of all 35 eligible patients, 24 had gross motor developmental dysfunction with an incidence of 68.6% (24/35). PCIS in the dysfunctional group was significantly lower than that in the non- dysfunctional group (81.0 ± 89.09 vs 92.18 ± 5.17, *P* = 0.001). Use of vasoactive medications (58.2% vs 8.2%, *P* = 0.006), length of PICU stay (22.50 [range, 14.50–27.75] vs 9.00 [7.00–17.00], *P* = 0.029), days on sedatives (9.0 [7.0–13.0] vs 1.0 [0.3–2.0], *P* **=** 0.021), and IMV days (7.46 ± 5.34 vs 1.27 ± 2.83, *P* = 0.001) were significantly higher than those parameters in the non- dysfunctional group.

**Table 1:** Participants Study Cohort Characteristics

Characteristics	Dysfunction Group (*n* = 24)	Non- Dysfunction Group (*n* = 11)	*P*
Age, months (mean ± SD)	20.9 ± 12.4	15.6 ± 12.9	0.005
Male sex (%)	58.3 (14/24)	63.6 (7/11)	0.766
Reason for admission (%)			
Cardiovascular	8.3 (2/24)	9.1 (1/11)	0.941
Respiratory	83.3 (20/24)	81.8 (9/11)	0.912
Gastrointestinal	8.3 (2/24)	9.1 (1/11)	0.941
PCIS (mean ± SD)	81.08 ± 9.09	92.18 ± 5.17	0.001
PICU length of stay, d, median (IQR)	22.50 (14.50–27.75)	9.00 (7.00–17.00)	0.029
Use of vasoactive medications, n (%)	58.2 (14/24)	8.2 (9/11)	0.006
IMV days (mean ± SD)	7.46 ± 5.34	1.27 ± 2.83	0.001
Sedative days, d, median (IQR)	8.50 (2.50–13.75)	2.0 (1.0–7.0)	0.021
Methylprednisolone, d, median (IQR)	5.5 (4.25–7.75)	3.00 (0.00–7.00)	0.130
CRRT days, d, median (IQR)	3.50 (0.00–7.00)		
Sepsis (%)	20.8 (5/24)	9.1 (1/11)	< 0.001
Surgical, n (%)	8.3 (2/24)		

*IQR* interquartile range, *SD* standard deviation, *PCIS* pediatric critical illness score, *CRRT* continuous renal replacement therapy, *IMV* invasive mechanical ventilation, *GMQ* gross motor quotient

### Linear correlation between GMQ and PCIS, length of PICU stay, IMV days, days on sedatives, days on methylprednisolone, and CRRT days

**Fig. 2:**
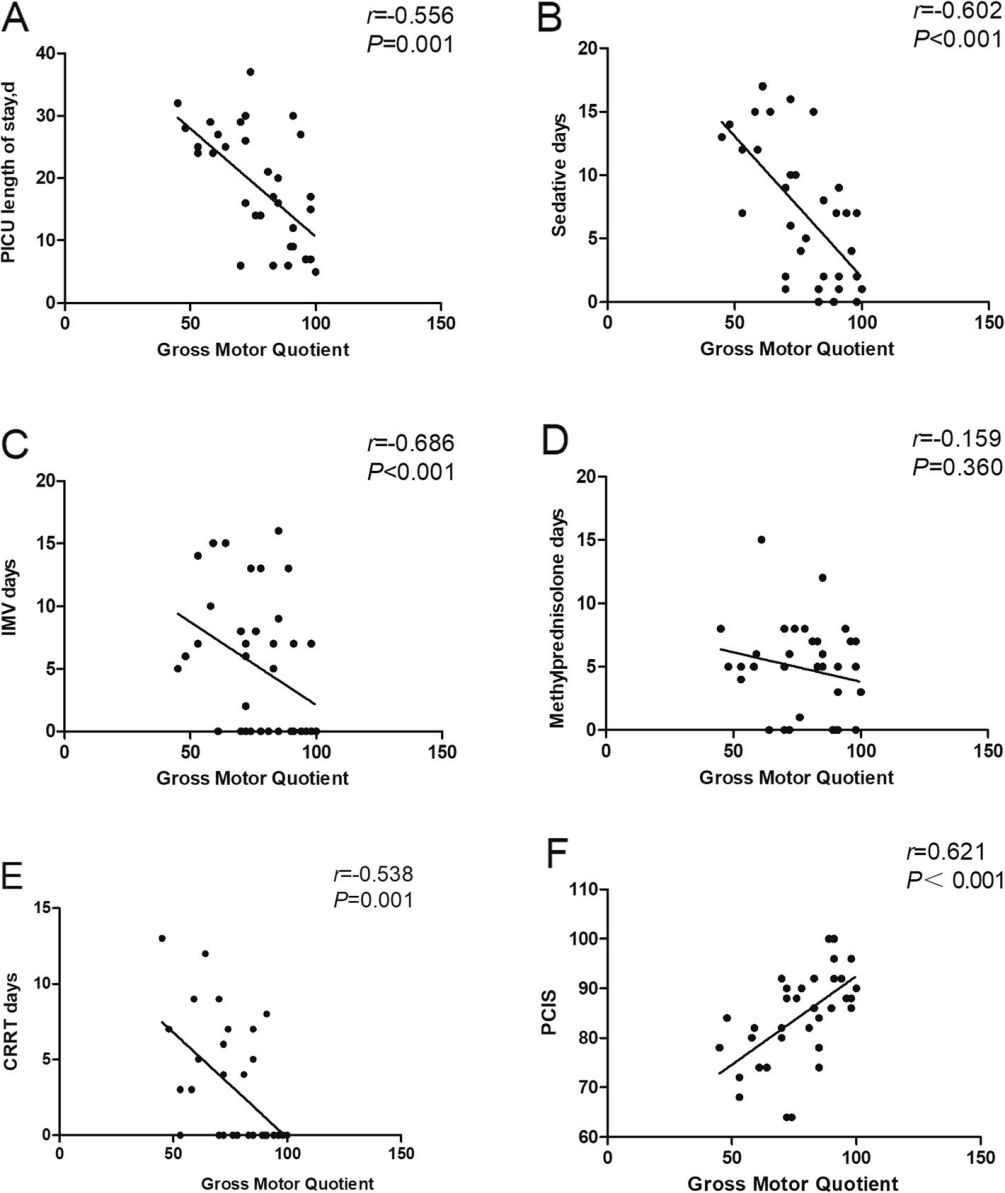
**a–f** Spearman’s correlation test was used to evaluate the relationships between GMQ and length of PICU stay, days on sedatives, IMV days, days on methylprednisolone, CRRT days and PCIS, respectively

We performed a linear correlation analysis between the GMQ and PCIS, length of stay in the PICU, IMV days, days on sedatives, days on methylprednisolone, and CRRT days. The results showed that GMQ was positively correlated with PCIS (*r* = 0.621, *P* < 0.001), while length of PICU stay (*r* = − 0.556, *P* = 0.001), days sedated (*r* = − 0.602, *P* < 0.001), IMV days(*r* = − 0.686, *P* < 0.001), and CRRT days (*r* = − 0.538, *P* = 0.001) were negatively correlated with the GMQ (Fig. 2a–f).

### Linear regression analysis between GMQ and length of PICU stay, days on sedatives, IMV days, CRRT days, PCIS, use of vasoactive medications, and sepsis

We performed a linear regression analysis of the above factors and the percentages of sepsis and vasoactive drugs with the GMQ. The results showed that sepsis (β = − 18.11, *P* = 0.003), PCIS (β = 0.55, *P* = 0.021) and IMV days (β = − 0.736, *P* = 0.001) were independent risk factors for a decreased GMQ (Table 2).

**Table 2:** Linear regression analysis of risk factors associated with GMQ

Factors	Gross Motor Quotient		
	β	Se(β)	*P*
IMV days	−0.736	0.260	0.001
Sepsis	−18.110	0.414	0.003
PCIS	0.550	0.338	0.021

The risk factors included in the linear regression analysis were length of PICU stay, days on sedatives, IMV days, CRRT days, PCIS, use of vasoactive medications and sepsis

## Discussion

The infant and toddler periods are vital times for gross motor development in humans. Gross motor behavior is one of the earliest directly observable elements of adaptive function. At 12 months old, children begin learning to walk, and their functional motor connectivity is correlated with walking [28]. During the toddler years, children change quickly in their motor function and physical growth, and their motor skills and ability to explore their environment improve [29]. Therefore, at this stage, factors such as diseases, environment and nutrition, interfere with the chances of infants and young children receiving external information, which affects their motor development. Uzark et al. [30] found that gross motor impairments were common in infants after cardiac operations. Friedman et al. [31] showed that young (aged 1–3 years) congenital diaphragmatic hernia survivors continued to have a high incidence of motor and language problems. Although motor developmental dysfunction presents in these populations, the motor developmental function levels of children in the PICU remain unclear. In addition, children in PICUs are more critically ill, undergo more invasive procedures, and receive more drugs; therefore, the level of motor developmental function in these infants and toddlers deserves attention. Unfortunately, few studies have focused on infants and toddlers in PICUs. This study was the first to assess gross motor developmental function in PICU survivors.

In this study, the incidence of gross motor developmental dysfunction was 68.6%. This statistic is higher than that reported for children after cardiac surgery (incidence 21–64%) [30, 32]. The incidence of motor dysfunction was 60% in infants who survived congenital diaphragmatic hernia repair [31], possibly because of the longer mechanical ventilation time, ICU stay, and more complications in infants and toddlers in the PICU compared with those with post-cardiac surgery. We believe that for critically ill children aged 1 month to 3 years, assessing gross motor developmental function is more important in guiding subsequent rehabilitation.

We also found that IMV days were significantly longer in the dysfunctional group than in the non- dysfunctional group. This suggests that the length of IMV is related to the occurrence of gross motor developmental dysfunction. IMV is one of the most commonly used treatment methods in PICUs, but it causes many dysfunctions in motor skills, cognition and psychology despite saving the lives of critically ill children. At present, many studies in adults have shown that IMV is a high-risk factor for ICU-acquired weakness (AW) [33, 34]. Patel et al. [35] found that patients with ICU-AW had significantly longer mechanical ventilation times. A systematic review of published work showed evidence of ICU-AW in 46% (95% confidence interval [CI] 43–49%) of adult ICU patients who experienced lengthy mechanical ventilation, sepsis, or multiorgan failure [36]. However, the effects of mechanical ventilation on infants’ gross motor developmental levels remain unreported. Our research showed that IMV days were associated with infants’ gross motor developmental dysfunction. IMV is also accompanied by longer PICU stays, more sedative use, more severe protopathic conditions and more invasive examinations. Length of PICU stay, days on sedatives, and days using CRRT were longer, and the vasoactive drug use rate, sepsis incidence and PCIS scores were higher in the dysfunctional group than in the non-dysfunctional group. These factors may promote gross motor developmental dysfunction in children.

Our research showed that the above factors were linearly correlated with GMQ, and the degree of gross motor dysfunction was significantly negatively correlated with PICU stay, sedative use and CRRT days and positively associated with PCIS scores. The GMQ of septic patients was also significantly lower than that of aseptic patients. To further analyze the independent risk factors that lead to gross motor developmental dysfunction, we performed a linear regression analysis of the above factors. The results showed that IMV days, sepsis and PCIS are independent risk factors for gross motor developmental dysfunction in PICU infants and toddlers. This is consistent with the results of an adult study on ICU-AW. Jongheet al [37]. conducted a multicenter, prospective study that showed that physical dysfunction in ICU patients was associated with prolonged mechanical ventilation. A prospective cohort study by Borges et al. indicated that physical activity, exercise capacity, and muscle strength were significantly reduced in ICU sepsis survivors, even at 3 months after discharge [38]. A meta-analysis conducted by Yang et al. incorporating 14 studies, showed that sepsis (OR, 2.2; 95%CI, 1.30–3.71) and duration of IMV (OR, 1.1; 95%CI, 1.00–1.22) were significantly associated with ICU-AW [39]. A multicenter study by Choong et al. suggested that Pediatric Risk of Mortality III (PRISM III) is an independent risk factor of social/cognitive dysfunction [40].

To investigate the independent risk factors for gross motor dysfunction in infants and Toddlers, we used a multivariate linear regression analysis. The result showed that IMV days, sepsis and PCIS were independent risk factors for gross motor developmental dysfunction. This is similar to the results of several adult studies [41, 42]. We believe that the above risk factors leading to children’s gross motor developmental dysfunction may have three pathways: 1. Prolonged mechanical ventilation and sepsis can lead to limb muscle atrophy [43, 44], resulting in weakened muscle strength in children; thus, abnormal assessment results may be due to weakened muscle strength; 2. Studies have confirmed that sepsis and IMV can cause brain dysfunction [45, 46]. However, the central nervous systems of infants and young children remain in the developmental stage, and motor neuron integrity is crucial to children’s mastering their motor skills. Therefore, damage to the child’s motor center due to sepsis and IMV days may affect the overall motor developmental level. 3. Impairment of cognitive function is related to motor developmental dysfunction, and previous studies have confirmed the effects of sepsis and mechanical ventilation on cognitive function [43, 47, 48]. Impaired cognitive function factors can affect motor function in children, especially infants [49, 50]. Whether this phenomenon exists in infantile and toddler PICU survivors requires further study.

This study had several limitations. First, the study was an observational study with a small sample size. Therefore, we found that only IMV days, sepsis and PCIS differed statistically when performing linear regression analyses on related factors. Previous studies showed that hormone and sedative use were significantly associated with the occurrence of physical dysfunction in pediatric and adult patients [51–53]. This study yielded inconsistent results; Therefore, more samples should be included for further analysis. Second, two assessment methods were used to assess the same patient pre-PICU and post-PICU. Due to the children’s pre-admission GMQs were unavailable, only the ASQ-3 questionnaire could be used to indirectly reflect gross motor function. Third, our study only assessed the participants’ motor development, while infant and toddler PICU survivors may have other developmental impairments such as cognitive, speech, psychological, and emotional disorders. Whether these dysfunctions are related to motor dysfunction warrants further study. Finally, we did not follow-up the enrollees to observe their gross motor function after discharge. In the next study, we will further follow this.

In conclusion, this study showed that gross motor developmental dysfunction in infantile and toddler PICU survivors are more common and may be exacerbated by experiences associated with longer IMV days and increasing illness severity combined with sepsis.

We suggest that early rehabilitative intervention in these children’s gross motor developmental function may reduce physical morbidity. Furthermore, detailed comprehensive investigations of developmental functions, including gross motor, fine motor, language, cognition and social abilities, are warranted.

## Data Availability

The datasets used and/or analyzed during the current study are available from the corresponding author on reasonable request.

## Abbreviations

ADLS: Activities of Daily Living Scale
ASQ-3: Age and Stages Questionnaires, Edition 3
CRRT: Continuous renal replacement therapy
GMQ: Gross motor quotient
ICU-AW: Intensive care unit-acquired weakness
IMV: Invasive mechanical ventilation
MRC: MedicalResearch Council
NICU: Neonatal intensive care unit
PCIS: Pediatric critical illness score
PDMS-2: Peabody Developmental Motor Scales, Second Edition
PICU: Pediatric intensive care unit
PRISM III: Pediatric Risk of Mortality III
